# Mechanisms of Osteoarthritis (OA) Pain

**DOI:** 10.1007/s11914-018-0477-1

**Published:** 2018-08-28

**Authors:** Terence W. O’Neill, David T. Felson

**Affiliations:** 1 Arthritis Research UK Centre for Epidemiology, Faculty of Biology, Medicine and Health, Manchester Academic Health Science Centre, The University of Manchester, The Stopford Building, Oxford Road, Manchester, M13 9PT UK; 2 NIHR Manchester Biomedical Research Centre, Manchester Academic Health Science Centre, Manchester University NHS Foundation Trust, Manchester, UK; 3 Salford Royal NHS Foundation Trust, Salford, UK; 4 Boston University School of Medicine, Boston, MA USA

**Keywords:** Osteoarthritis, Pain, Synovitis, Bone marrow lesions, Central sensitisation, Peripheral sensitisation

## Abstract

**Purpose of Review:**

Osteoarthritis (OA) is a major cause of pain and disability worldwide. There is, however, a relatively poor correlation between the severity of OA based on plain radiograph changes and symptoms. In this review, we consider the mechanisms of pain in OA.

**Recent Findings:**

It is now widely recognised that OA is a disease of the whole joint. Data from large observational studies which have used magnetic resonance imaging (MRI) suggest that pain in OA is associated with a number of structural factors including the presence of bone marrow lesions (BMLs) and also synovitis. There is evidence also of alterations in nerve processing and that both peripheral and central nerve sensitisation may contribute to pain in OA.

**Summary:**

Identification of the causes of pain in an individual patient may be of benefit in helping to better target with appropriate therapy to help reduce their symptoms and improve function.

## Introduction

Osteoarthritis (OA) is the most prevalent chronic joint disease and remains one of the few chronic disorders of ageing for which there is little effective treatment and none proven that delays disease progression. It can affect small, medium and large joints though in terms of painful disease, the knee is the most frequently affected with up to one in eight men and women over the age of 60 years having evidence of symptomatic knee OA [1, 2]. OA is a leading cause of disability in older adults and is one of the leading causes of impairment of mobility in the elderly population in the USA [3]. It accounts also for substantial direct health-care costs related largely to the requirement for joint replacement surgery in those with end stage disease [4].

Pain is the predominant symptom of OA and is what usually leads those affected to seek medical care. The pain in OA is typically aggravated by joint use and relieved by rest. It tends to be localised to the affected joint (s) though may occur beyond and in some cases may be referred, for example, pain may sometimes be experienced in the thigh/knee in patients with hip OA. In the early stages of disease, symptoms including pain are often intermittent becoming more frequent and severe as the disease progresses. It is widely recognised, however, that there is a poor correlation between the severity of disease based on plain x-ray changes and symptoms of pain [5, 6]. Asymptomatic radiographic OA is common at the finger and spine joints though may occur also in large weight bearing joints [7]. In an analysis of data from the National Health and Nutrition Examination Survey (NHANES 1), less than 50% of those with radiographic OA had knee pain [8]. The reason for this apparent discordance remains unclear though is likely in part because plain radiography is an insensitive indicator of the structural and nociceptive changes which occur in OA.

Identification of the sources and mechanisms of pain in OA is important; an understanding of the cause (s) of pain may help in better targeting affected individuals with appropriate therapy and may also potentially help identify alternative therapies to help reduce symptoms and improve function. In this review, we consider structural changes which occur in OA that are linked with pain and also neuronal mechanisms and alterations of pain processing which are involved in OA pain.

## Structural Changes in OA

The signature pathologic feature of OA is articular cartilage loss which is typically recognised on plain radiographs as a reduction in joint space. Loss of cartilage and joint disruption is linked with attempts at repair with new bone formation occurring and the development of subchondral sclerosis and osteophytes. With the advent of more detailed imaging studies, particularly magnetic resonance imaging (MRI), OA is now widely recognised as a disease involving the whole joint including ligaments, menisci, synovium (synovitis), and joint capsule [9]. MRI studies also show evidence of abnormal bone structure at the subchondral boundary with cysts and bone marrow lesions (BMLs)—the latter best visualised on MRI as hyper-intense areas using fat- suppressed T2-weighted or proton density-weighted imaging [10].

## Nociception Within the Joint

Within the joint, there are pain-sensing afferent neurons (nociceptors) in many of the anatomic tissues affected by OA including the periosteum and subchondral bone [11, 12], soft tissues including ligament insertions [13], menisci [14, 15], and synovium [16]. Although cartilage loss is an important structural feature, it is not innervated and therefore cannot be a direct source of pain in mild to moderate disease. In-vivo studies corroborate this. For example, in a study in which an orthopaedist underwent arthroscopy with only the soft tissues around the joint anaesthetised, probes inside the joint suggested that probes of the cartilage were not painful [17]. In more severe disease, neurovascular invasion at the osteochondral junction may occur and potentially contribute to pain [18]. Also, in mild and moderate disease, microscopic cartilage debris can be phagocytosed by cells lining the synovium, triggering inflammatory responses and pain from synovitis [19].

## Evidence for Pain/Structure Relationships

One approach to look for evidence that structural changes within the OA joint are linked with knee pain is to undertake MRI scans in those with and without pain to identify which structural findings are associated with pain [20–22, 23•]. Using data from such studies, pain has been associated with a number of structural factors including bone marrow lesions, synovial thickening (synovitis)/knee effusion, and periarticular lesions including anserine bursitis, though the latter were uncommon [20–22, 23•]. Other structural abnormalities have been suggested also to contribute to the occurrence of pain. Looking at these in turn:

- Bone Marrow Lesions

Bone marrow lesions (BMLs) are poorly circumscribed lesions below the subchondral bone which are observed on MRI in up to 80% of individuals with symptomatic OA [20]. On histopathology, BMLs are characterised by fat necrosis, localised marrow fibrosis and microfractures of the trabecular bone that are associated with active bone remodelling and repair [24, 25]. These lesions occur in areas where there is increased stress, for example where there is knee malalignment, and they almost certainly represent bone injury in areas of high focal loading.

In MRI studies, BMLs are seen more frequently in painful knees with OA than non-painful knees [23•]. For example, in a large observational study among individuals with radiographic OA and pain, 36% had large BMLs in their knees on MRI vs. only 2% of OA knees that were not painful (*p* < .001) [20]. Data from a number of prospective OA studies suggest that fluctuation in the size of these lesions correlates with the fluctuation of knee pain, suggesting that these lesions are a cause of pain [26–28]. Specifically, in the Multicenter Osteoarthritis Study where serial MRIs were obtained, it was reported that new onset of BMLs or their enlargement was strongly associated with new onset frequent knee pain in previously pain free knees [26]. In a later report from the study, Zhang [28] reported that a decrease in BML volume over a 30-month follow-up was strongly related to a reduction in knee pain. In addition to these observational data, there is evidence that targeted interventions to reduce loading in knee OA are linked with a reduction in BMLs and a reduction in knee pain [29]. These data provide good evidence to support the view that BMLs are a cause of pain in OA. The mechanism by which BMLs may cause pain is unknown though it may, in part, be related to the occurrence of subchondral microfractures.

(b) Synovitis

On arthroscopy, synovitis is seen in approximately 50% of the knees of patients with painful OA and in an even higher percentage using MRI [30, 31]. Loeuille [32] comparing the MRI, histologic and arthroscopic appearance of synovium in persons with symptomatic knee OA reported a high correlation between the degree of synovial thickening on MRI and macroscopic scoring of synovitis by an arthroscopist (*r* = 0.58). Thickness was also correlated with infiltration of inflammatory cells into the subsurface layers of synovium (*r* = 0.46).

Using non-contrast-enhanced MRI, which yields an incomplete view of synovitis, Hill and colleagues reported that synovitis was correlated cross-sectionally with the severity of knee pain in persons with knee OA [21]. In a further study Hill et al. found a modest correlation (*r* = 0.21, *p* = 0.0003) among 270 people with symptomatic knee OA and serial MRIs between *change* in synovitis observed on MRI with change in severity of knee pain over time [33]. Hill’s findings have been confirmed by Zhang and colleagues using data from serial MRI’s in the MOST Study who found that change in synovitis score was strongly related to change in pain—an increase in score being associated with an increase in knee pain [28]. Contrast enhances the appearance of synovium without showing the other surrounding structures and therefore yields a more accurate view of synovitis. Baker [34] reported, on a subset of the MOST cohort who had gadolinium-enhanced MRIs, that synovitis was more prevalent in the knee pain group even after adjustment for age, sex, BMI and BML’s. Those with a lot or extensive synovitis constituted less than 20% of the sample, but those with this level of synovitis had 4.8 times the odds of mild pain (vs no pain) and had a marked increased risk of moderate to severe knee pain (odds ratio = 9.2) compared with those without synovitis. Even among persons without radiographic osteoarthritis (many of whom have some cartilage loss on their MRI suggesting early disease), extensive synovitis was present in a subsample and was associated with a marked increase in the risk of knee pain [34].

Further supportive evidence of a role for synovitis in explaining knee pain derives from an open-label trial of steroid therapy in 120 men and women who had contrast-enhanced scans prior to and following an intra-articular steroid injection (80 mg) [35•]. After 2 weeks, those whose pain improved had a more marked reduction in synovitis compared to those who did not respond; furthermore, among those whose pain subsequently recurred within 6 months, both pain and synovial tissue volume increased. The exact mechanism by which synovitis may mediate pain is unclear though may act through the production of cytokines/inflammatory mediators that sensitise or activate sensory nerves, or by mechanical stimulation of nociceptors by thickened synovium, effusion or both.

(c) Other

Cartilage is aneural and does not have pain fibres and so change in cartilage is not directly linked with the occurrence of pain; this is confirmed in longitudinal studies which suggest that cartilage loss and pain relief are poorly, if at all, correlated [36]. However, it was reported that a lesion in which cartilage is denuded to bone (with subchondral bone plate exposure) is associated with prevalent and incident knee pain in patients with knee OA [37]. Bone attrition observed as flattening or depression of the articular cortex has been associated with pain though it often occurs with other OA features associated with pain including BMLs [38, 39]. There is variable evidence related to meniscal tears. Ashraf suggested that meniscal pathology may contribute to pain [15]. In a separate study, an association between meniscal damage and knee pain disappeared after taking into account the severity of the underlying OA [40]. Tears of the ACL (identified by MRI) are quite common in OA though they are not especially associated with the occurrence / progression of pain [41].

## Neuronal Pathways and Pain Processing

Nociceptors in the OA joint may be stimulated by a variety of noxious stimuli including physical/mechanical or chemical stimuli including inflammatory mediators such as bradykinins & prostaglandin E2. There are several types of receptors on nociceptors that transduce noxious stimuli to pain (e.g., ASICs, TRPs), though the transient receptor potential (TRP) family is probably the best characterised and possess transmembrane domains which function as ion channels on stimulation [42].

The action potential generated on stimulation of nociceptors on peripheral nerve terminals is transmitted through unmyelinated C nerve fibres and myelinated Aδ fibres. Due to their faster conduction, Aδ fibres are responsible for the transmission/experience of sharp pain while type C fibres respond to multiple signals and are responsible for the sensation of more diffuse burning pain [43].

Afferent pain fibres transmit pain inputs from the joint to the spinal cord where synaptic processing occurs and then in ascending pathways to the thalamus and higher centres in the somatosensory cortex. Descending fibres in the spine help to modulate the pain inputs. Pain is also strongly influenced by a range of socio-cultural and psychological factors, including underlying anxiety and depression. The term ‘neuromatrix’ has been coined to reflect the complex interplay between these factors and the underlying neurologic architecture [44].

In response to injury, or inflammation or other nociceptive stimuli, the threshold for local nerve excitation and transmission of signals may be lowered and lead to increased responsiveness of the peripheral nociceptors, a phenomenon which is termed ‘peripheral sensitisation’ and which may explain in some people with knee OA the phenomenon of hyperalgesia (or hypersensitivity) and allodynia (pain in response to a normally non-noxious stimuli).

Recent evidence suggests an important role for nerve growth factor (NGF) in mediating inflammatory pain by increasing nociceptor sensitivity [45–47]. In an animal model of arthritis produced by injection of complete Freund’s adjuvant into the knee joint, systemic administration of anti-NGF attenuated nerve fibre sprouting and arthritis pain [48]. Nerve growth factor blockade has been shown also in human studies to reduce pain in knee and hip OA [49, 50•, 51], indicating a causal link between NGF and OA pain.

Similar to peripheral sensitisation, central nervous system pain pathways including those governing descending inhibition may be affected by persistent stimulation of dorsal root ganglia from inflammatory cytokines originating in the joint and leading to central sensitisation. Evidence points to the presence of both peripheral and central nervous system sensitization as sources of pain in osteoarthritis [52–55]. Sensitization tends to occur later in the disease and may explain why osteoarthritic pain may become more severe and continuous. Sensitisation may explain also why some people may be more resistant to standard analgesic treatment and also the efficacy of centrally active agents such as duloxetine [56].

## Summary

OA is a disease of the whole joint including bone, cartilage and synovium. MRI has been a very valuable tool in understanding the underlying structural correlates of pain in those with symptomatic OA and data from observational and intervention studies using MRI has helped characterise the contributions particularly of synovial inflammation (synovitis) and bone marrow lesions in explaining pain. There is evidence also that altered sensitivity of the nerves which supply the knee or within the CNS may explain more persistent pain in OA. Current analgesics in OA are limited either by lack of effect or troublesome side effects. Identification of the cause(s) of pain in individual patients may be of benefit in helping to better target them with appropriate therapy to help reduce their symptoms and improve function.
